# Echinocandin B biosynthesis: a biosynthetic cluster from *Aspergillus nidulans* NRRL 8112 and reassembly of the subclusters *Ecd* and *Hty* from *Aspergillus pachycristatus* NRRL 11440 reveals a single coherent gene cluster

**DOI:** 10.1186/s12864-016-2885-x

**Published:** 2016-08-08

**Authors:** Wolfgang Hüttel, Loubna Youssar, Björn A. Grüning, Stefan Günther, Katharina G. Hugentobler

**Affiliations:** 1 Pharmaceutical Chemistry, Institute of Pharmaceutical Sciences, University of Freiburg, Albertstr. 25, 79104 Freiburg, Germany; 2 Present address: Institute for Environmental Health Sciences and Hospital Infection Control Medical Center, University of Freiburg, Breisacher Strasse 115b, 79106 Freiburg, Germany; 3 Pharmaceutical Bioinformatics, Institute of Pharmaceutical Sciences, University of Freiburg, Hermann-Herder-Str. 9, 79104 Freiburg, Germany; 4 Present address: Bioinformatics Group, Department of Computer Science, University of Freiburg, Georges-Köhler-Allee 106, 79110 Freiburg, Germany

**Keywords:** Nonribosomal peptides, Biosynthesis, *Aspergillus* section *Nidulantes*, Fungi, Sequence analysis

## Abstract

**Background:**

Echinocandins are nonribosomal lipopeptides produced by ascommycete fungi. Due to their strong inhibitory effect on fungal cell wall biosynthesis and lack of human toxicity, they have been developed to an important class of antifungal drugs. Since 2012, the biosynthetic gene clusters of most of the main echinocandin variants have been characterized. Especially the comparison of the clusters allows a deeper insight for the biosynthesis of these complex structures.

**Results:**

In the genome of the echinocandin B producer *Aspergillus nidulans* NRRL 8112 we have identified a gene cluster (*Ani*) that encodes echinocandin biosynthesis. Sequence analyses showed that *Ani* is clearly delimited from the genomic context and forms a monophyletic lineage with the other echinocandin gene clusters. Importantly, we found that the disjunct genomic location of the echinocandin B gene cluster in *A. pachycristatus* NRRL 11440 on two separate subclusters, *Ecd* and *Hty*, at two loci was likely an artifact of genome misassembly in the absence of a reference sequence. We show that both sequences can be aligned resulting a single cluster with a gene arrangement collinear compared to other clusters of *Aspergillus* section *Nidulantes*. The reassembled gene cluster (*Ecd*/*Hty*) is identical to a putative gene cluster (*AE*) that was previously deposited at the NCBI as a sequence from *A. delacroxii* NRRL 3860. PCR amplification of a part of the gene cluster resulted a sequence that was very similar (97 % identity), but not identical to that of *AE*.

**Conclusions:**

The Echinocandin B biosynthetic cluster from *A. nidulans* NRRL 8112 (*Ani*) is particularly similar to that of *A. pachycristatus* NRRL 11440 (*Ecd*/*Hty*). *Ecd*/*Hty* was originally reported as two disjunct sub-clusters *Ecd* and *Hty*, but is in fact a continuous sequence with the same gene order as in *Ani*. According to sequences of PCR products amplified from genomic DNA, the echinocandin B producer *A. delacroxii* NRRL 3860 is closely related to *A. pachycristatus* NRRL 11440. A PCR-product from the gene cluster was very similar, but clearly distinct from the sequence published for *A. delacroxii* NRRL 3860 at the NCBI (No. AB720074). As the NCBI entry is virtually identical with the re-assembled *Ecd*/*Hty* cluster, it is likely that it originates from *A. pachycristatus* NRRL 11440 rather than *A. delacroxii* NRRL 3860.

**Electronic supplementary material:**

The online version of this article (doi:10.1186/s12864-016-2885-x) contains supplementary material, which is available to authorized users.

## Background

**Fig. 1:**
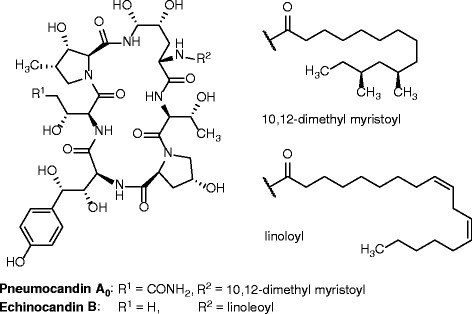
Structures of Pneumocandin A and Echinocandin B, two important members of the echinocandin family

Echinocandins are fungal nonribosomal cyclic hexapeptides conjugated with a fatty acid or highly reduced polyketide side chain (Fig. 1).

They have a strong antifungal activity because they specifically inhibit β-1,3-glucan synthase, the enzyme responsible for the main polymer in cell wall biosynthesis. Semi-synthetic derivatives have become important antifungal drugs for treatment of invasive mycoses [1–3]. Besides their pharmacological properties, their extraordinary structures offer numerous challenging questions for biosynthetic studies. Depending on the individual echinocandin, four or five of the six amino acids are non-proteinogenic. Most amino acid modifications are due to hydroxylations catalyzed by cytochrome P450 monoxygenases or non-heme dioxygenases. However, in two amino acids, 3-hydroxy-4-methylproline and dihyroxyhomothyrosine, the carbon skeleton is also non-canonical. The peptide ring of echinocandins is closed by an N-acyl-hemiacetal moiety, which is very sensitive to hydrolysis as a rule. Nevertheless, in echinocandins it is conformationally stabilized, so that the cyclopeptides can be handled without protection. The first systematic studies on echinocandin biosynthesis were carried out the early 1990s when researchers from Merck & Co. Research Laboratories identified the biosynthetic building blocks of the pneumocandin through ^13^C-labeling experiments and developed a comprehensive concept of pneumocandin biosynthesis [4, 5]. However, it was not until two decades later that echinocandin biosynthesis was elucidated at the genetic level [6, 7]. In the genome of *Aspergillus pachycristatus* NRRL 11440 [8], previously named as *Emericella rugulosa*, two separate partial clusters were located on different contigs of the assembly and were found to be responsible for encoding the synthesis of echinocandin B. The larger section, *Ecd*, encoded most of the enzymes required for echinocandin assembly and decoration. This included genes for a nonribosomal peptide synthetase (NRPS) with domains for the assembly of six amino acids and peptide cyclization, an Acyl Amp ligase for acylation with linoleic acid, three oxygenases, and an ABC transporter. The other cluster, *Hty*, contained the genes for the homotyrosine biosynthetic pathway and two additional oxygenases. The functions of many of the biosynthetic enzymes encoded by *Ecd* were demonstrated experimentally [6, 7]. In 2014, we described an α-ketoglutarate/Fe^2+^-dependent proline hydroxylase involved in pneumocandin biosynthesis in *Glarea lozoyensis* [9]. More recently, the functions of four oxygenases in *G. lozoyensis* cluster *GL* were demonstrated by deletion in the host strain [10, 11]. The results were consistent with and complemented the findings for *A. pachycristatus*.

Taking all these results together, the nearly complete echinocandin biosynthesis can be modelled, though the precise order of some of the oxidation steps remain to be confirmed.

In the meantime, the sequences of additional echinocandin biosynthetic clusters were deposited at the NCBI by researchers from Toyama Prefectural University (Table 1). These sequences now allow phylogenetic reconstructions and more sound investigations of sequence-structure relationships and [12, 13].

**Table 1:** Currently known echinocandin biosynthesis clusters [12, 13]

Cluster acronym	Organism (synonyms)	Echinocandin Product	NCBI-ID
*AA*	*Aspergillus aculeatus* ATCC 16872	aculeacin [27]	GOLD Project ID: Gp0010055
*AM*	*Aspergillus mulundensis* Y-30462 = DSMZ 5745 (= *Aspergillus sydowii* var. *mulundensis*)	mulundocandin [28]	KP742486
*Ani*	*Aspergillus nidulans* NRRL 8112 (= *Emericella nidulans* DSM 946)	echinocandin B	KT806042
*CC*	*Coleophoma crateriformis*	antibiotic WF738A	AB720076
*CE_1*	*Coleophoma empetri* F-11899	antibiotic WF11899A-C	AB723722
*CE_2*	*Coleophoma empetri* No. 14573	antibiotic WF14573A,B	AB720725
*Ecd/Hty* (identical with *AE*)	*Ecd/Hty*: *Aspergillus pachycristatus* NRRL 11440 (= *Emericella rugulosa*, *Aspergillus nidulans* var. *roseus* ATCC 58397) *AE*: stated strain: *Aspergillus delacroxii* NRRL 3860 (= *Emericella nidulans* var. *echinulatus*	echinocandin B	*Ecd/Hty*: assembly of *Ecd* JX421684 and *Hty* JX421685 *AE*: AB720074
*GL*, *Glo*	GL: *Glarea lozoyensis* wildtype strain ATCC 20868 Glo: *G. lozoyensis* mutant strain ATCC 74030	pneumocandin	NW_007360987, AGUE01000179
*PH*	*Phialophora cf. hyalina* No. 16616 (=*Tolypocladium parasiticum*)	antibiotic WF16616	AB720726

Here we present the echinocandin B gene cluster from *A. nidulans* NRRL 8112 (*Ani*) identified by whole genome sequencing and sequence analysis. The *Ani* gene cluster is compared with the known echinocandin biosynthesis clusters from *Aspergillus* section *Nidulantes*. We also found that the two separated semi-clusters *Ecd* and *Hty* encoding echinocandin B biosynthesis in *A. pachycristatus* can be aligned into a continuous sequence (*Ecd/Hty*), which is virtually identical with the echinocandin B biosynthesis cluster *AE* deposited at the NCBI as a sequence from *A. delacroxii* NRRL 3860 (= *E. nidulans* var. *echinulata*).

## Results and discussion

### Echinocandin biosynthesis cluster from *A. nidulans* NRRL 8112

*A. nidulans* strain NRRL 8112 was originally isolated from India and was described as a weak producer of echinocandin B in a patent from Eli Lilly in 1976 (3.5 g/200 L culture broth) [14]. As several other *A. nidulans* strains are well characterized and often used for molecular genetic research, we thought that echinocandin B biosynthesis by this strain would be of particular interest. To identify the corresponding cluster from *A. nidulans*, genomic DNA of NRRL 8112 was sequenced using the Illumina technique. Originally a reference assembly with the genome of *A. nidulans* FGSC A4 (assembly ASM14920v1) was attempted, however, more than one million reads remained unmapped. As an alternative, the reads were assembled *de novo* resulting a genome of 31.6 Mbp partitioned in 1,572 contigs. By BLASTn search using genes of the *Ecd* biosynthetic cluster as query sequences we localized an echinocandin biosynthesis cluster of about 51 kb (*Ani*) in a relatively small contig (56 kbp). The set of genes in *Ani* is equivalent to that of echinocandin B biosynthesis cluster *AE* (≙ *Ecd*/*Hty*) and the mulundocandin biosynthesis cluster. The gene orders are fully collinear with those from *Aspergilli* depicted previously [12].

### Determination of terminal regions by comparison with *AE* from *A. delacroxii*

A comparison with the currently known echinocandin biosynthetic clusters (Table 1) revealed a highly conserved “core” set of genes, most of them encoding enzymes required for biosynthesis. Though evidence was rather vague, possible genes in the periphery of the core cluster were considered to be linked to echinocandin biosynthesis [7, 15]. However, similar genomic context in the flanking regions of the clusters indicating a function for echinocandin biosynthesis was only found in two closely related clusters from *Coleophoma empetri* strains (*CE_1* and *CE_2* in Table 1) [12].

**Fig. 2:**
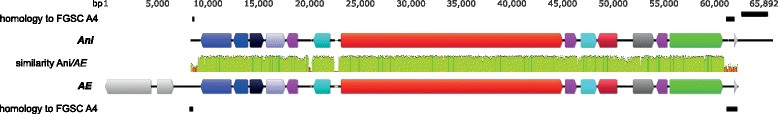
Schematic representation of a DNA sequence alignment of the echinocandin B biosynthesis clusters and flanking regions of the echinocandin gene clusters *Ani* and *AE*. Pairwise sequence identities are depicted as columns showing the overall identity about 50 bp. The following color code is used for sequence identity: dark green: 100 %; *bright-green*: 30 % – <100 % and *red:* <30 %. The *black* bars indicate segments with homology to sections in the genome of *A. nidulans* FGSC A4 determined by Discontiguous Megablast (e-values <10^−130^)

The gene order in *Ani* was identical to that in the *AE*-cluster*,* which was deposited at the NCBI as a sequence from *A. delacroxii* NRRL 3860*,* previously named as *E. nidulans* var. *echinulata* (Table 1) [16–18]. This allowed to align the DNA-sequences of the clusters over the whole lengths including the right- and left-flanking regions (Fig. 2). For the region considered as the “core” cluster, the average nucleotide sequence identity is about 89 %. At both ends of this region it drops abruptly to about 30 %. A BLAST analysis revealed that the left- and right-flanking regions comprised a genomic context orthologous to sections in the genome of *A. nidulans* FGSC A4 (ATCC 38163), a strain that lacks the echinocandin cluster. Thus*,* even in closely related species, echinocandin gene clusters appear as discrete genomic units and lack similarity in the near genomic neighborhoods [12]. This observation strongly suggests that genes in close proximity to the “core” cluster are not involved in echinocandin biosynthesis. As the available DNA-segments encoding echinocandin B biosynthesis were relatively short, an analysis of more distant regions was not possible.

### Comparison of cluster *AE* with *Ecd* and *Hty*

**Fig. 3:**
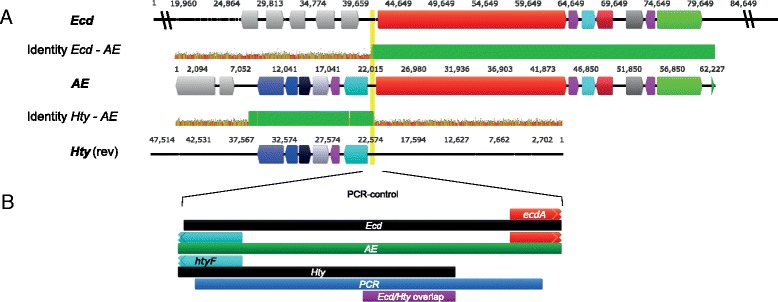
**a** Schematic representation of a reference alignment of *Ecd* and *Hty* to *AE*. Pairwise sequence identities are depicted as bars between the sequences. One bar is define by about 50 bp. The following color code is used for sequence identity: *green* = 100 %; *greeny-brown* 30 % – <100 % and red <30 %. The *yellow* mark indicates a region of approximately 400 bp in which *Ecd* and *Hty* are highly similar (96 % identity). **b** Excerpt showing the overlapping region (*purple*) and the section verified by sequencing of PCR product (*blue*)

**Fig. 4:**
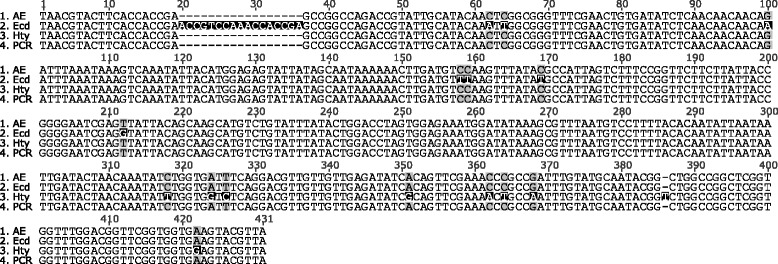
Overlapping region of *Ecd* and *Hty* aligned with the PCR product amplified from genomic *A. pachycristatus* DNA. (An alignment with the entire PCR-product as outlined in 2b is shown in Additional file 1: Figure S3)

In contrast to all other clusters, echinocandin B biosynthesis by *A. pachycristatus* was reported to be split in two separate clusters, *Ecd* and *Hty,* which were found on different contigs of the genome assembly [7]. A minimal chromosomal distance of at least 42.5 kb was estimated. When we compared genes of the *AE*-cluster with those from *Ecd* and *Hty*, we found strikingly high sequence identities (>95 %). As the gene orders in *Ecd* and *Hty* are collinear with that of *AE*, both half-clusters could be aligned to *AE* (Fig. 3). Notably, there was a coinciding section of about 400 bp between *Ecd* and *Hty* (96.0 % sequence identity) (Fig. 4). This overlap was used to assemble both partial clusters into a continuous sequence (*Ecd*/*Hty*). To confirm the overlap of *Ecd* and *Hty*, this area and the flanking regions were amplified by PCR from *A. pachycristatus* genomic DNA (Fig. 3b and Additional file 1: Figures S1–S3 and Table S1). The PCR-product of the 5′-terminus proved to be identical with *Hty* and contained a part of the cytochrome-P450 oxygenase gene *HtyF*. At the 3′-terminus, a section of *Ecd* up to the NRPS-gene (*EcdA*) was found. In contrast, PCR experiments that aimed at the amplification of *Hty* and *Ecd* as separate clusters, were unsuccessful. The gene arrangement in the fused cluster *Ecd/Hty* was identical with that in *AE*, *Ani* and *AM* (cf*.* Table 1). Notably, the sequence identity between *Ecd/Hty* and *AE* was 99.9 % over the full lengths of the alignment. Furthermore, most of the non-matching sites were due to gaps and ambiguities in one of the sequences. Only three base pairs in the 53.9 kbp alignment were genuinely inconsistent (99.99 % sequence identity). The near complete sequence identity strongly suggests that the same strain has been used in both sequencing projects. However, according to the literature, the strains originated from unrelated sources. *A. delacroxii* (A-32204, NRRL 3860) was first described by researchers from the Ciba-Geigy AG, Switzerland [16, 17] as *A. nidulans* var. *echinulatus*. It was originally isolated from beech leaves at a non-specified place. *A. pachycristatus* NRRL 11440 (formerly *E. rugulosa*, ATCC 58397) was isolated by researchers of Eli Lilly from a soil sample from Greenfield, Indiana. It was originally classified as *A. nidulans* var. *roseus* [19]. Comprehensive experimental studies reported in the literature and our PCR-control of genomic DNA confirmed that strain ATCC 58397 actually has a cluster of the reported sequence. To find out more about the relationship of the strains, we first amplified and sequenced the internal transcribed spacers (ITS) region of both strains [20]. The resulting sequences differed only in one basepair. A large number of ITS sequences with 99 % identity was found at the NCBI Nucleotide database. Most of them were from *A. nidulans* strains, but some also from *A. pachychristatus* (*E. rugulosa*) and *A. delacroxii* (*E. echinulata*) (Additional file 1: Table S2 and Figure S5). As the ITS DNA marker was not sufficient to differentiate the strains, a part of the calmoludin gene was analyzed [8, 21]. In an alignment of calmodulin gene sequences from *A. delacroxii* and *A. pachycristatus* strains deposited at the NCBI Nucleotide database more than 30 species-conserved differences were found (Additional file 1: Figure S6), which allows a clear differentiation of both. This was also visualized in a phylogenetic tree based on the calmodulin sequences of about 120 *Aspergillus* (*Emericella*) strains, in which the *A. delacroxii* and *A. pachycristaus* strains were located in distinct subclades (Additional file 1: Figure S7)*.*

Surprisingly, the calmodulin sequence of *A. delacroxii* NRRL 3860 (Additional file 1: Figure S8) differed in only two basepairs from that of *A. pachycristatus* NRRL 11440. Both sequences could be clearly assigned to the *A. rugulosa/A. pachychristatus* group rather than to the *E. delacroxii* (*E. echinulata*) strains (Additional file 1: Figure S7).

In addition to the common genomic marker sequences, a region between the NRPS and the adjacent cytochrome p450 oxygenase gene of the echinocandin biosynthesis cluster was analyzed. It was the same region which had already been sequenced from *A. pachycristatus* to confirm the connectivity of *Ecd* and *Hty*. As for the calmodulin fragments, the sequences of *A. delacroxii* and *A. pachychrytatus* (and the *AE* cluster) were very similar, but clearly distinct (97 % identity, see Additional file 1: Figure S10). From these results, we concluded that the *AE* cluster most likely originates from *A. pachycristatus* NRRL 11440 and not from *A. delacroxii* NRRL 3860.

So far, echinocandin biosynthesis has been found only in relatively narrow phylogenetic regions of two related lineages of fungi, the Aspergillacea (Eurotiomycetes) and Helotiales (Leotiomycetes) [12]. Although these clades diverged an estimated 290 – 390 million years ago [22], echinocandin biosynthesis clusters share a remarkable degree of similarity. The sequence identities of *Ani* proteins with their orthologues range from 48 to 94 %. The only exception is *AniJ*, a putative protein of unknown function (31–85 %), whose orthologs are generally more diverse. Recently, Bills, An and coworkers have published a detailed phylogenetic analysis of the currently known echinocandin biosynthetic clusters [12]. The phylogenetic trees of individual pathway proteins were monophyletic. The degree of sequence divergence in the echinocandin pathway genes was similar to that in genes involved in primary carbohydrate metabolism, thus strongly suggesting a monophyletic lineage of echinocandin biosynthesis clusters originating from a common ancestor of the Aspergillaceae and Helotiales. No evidence was found for horizontal gene transfer at later evolutionary stages. Table 2 shows the sequence identities of *Ani* proteins with their orthologues in the other clusters. It reveals consistent trends depending on the individual cluster and the type of protein. No significant deviations were found in *Ani* proteins. Thus, *Ani* conforms well to the concept of a monophyletic lineage for echinocandin biosynthesis. The closest clusters to *Ani* were *Ecd/Hty* from *A. pachycristatus* and *AM* from *A. mulundensis* with 91 % and 83 % overall identity, respectively.

**Table 2:**
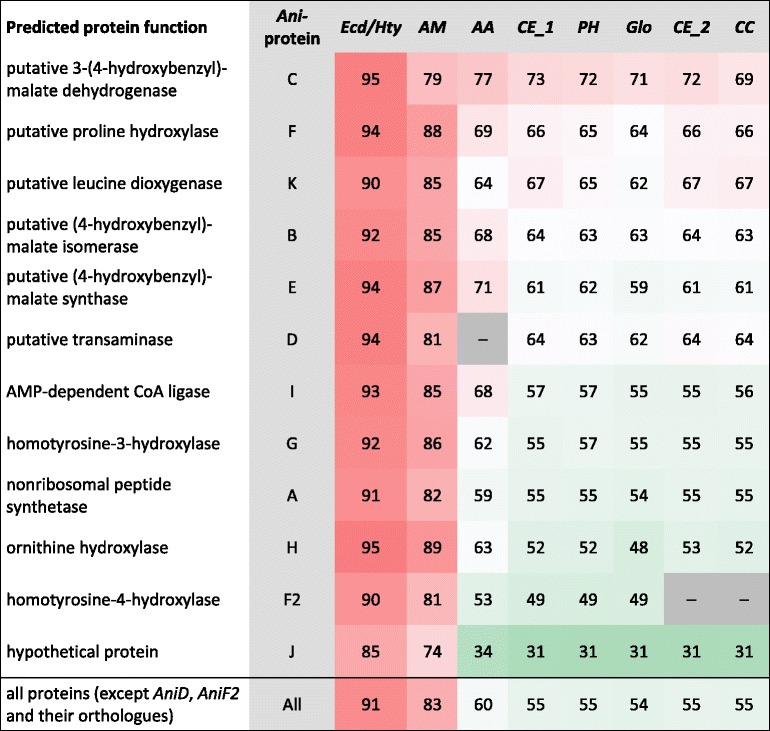
Sequence identity of *Ani-*proteins to their orthologs in other echinocandin biosynthetic clusters. Color shading was used to visualize sequence identities. Color code: red: 100 %, white: 50 % and green 0 %. Protein sets: *Ani*: *A. nidulans* NRRL 8112, *AA*: *A. aculeatus* ATCC 16872, *CC*: *C. crateriformis, CE*_1: *C. empetri* F-11899, *CE*_2: *C. empetri*, *PH*: *T. parasiticum* No. 16616, *Ecd/Hty*: *A. pachycristatus* NRRL 11440, formerly *E. rugulosa*, *AM*: *A. muludensis* DSMZ 5745, *GL*: *G. lozoyensi* ATCC 20868, *Glo*: *G. lozoyensis* mutant strain ATCC 74030 (*GL* and *Glo* are identical except from two residues in protein *GloC* ≙ *GLOXY4*)

## Conclusions

In summary, we have identified the echinocandin B biosynthesis cluster *Ani* in the genome of the echinocandin B producer strain *A. nidulans* NRRL 8112. The gene order in *Ani* is collinear with that in the other known clusters of *Aspergilli* section *Nidulantes* (*AE* ≙ *Ecd*/*Hty* and *AM*). Sequence comparison of the individual proteins revealed that *Ani* forms a strictly monophyletic clade with the other echinocandin gene clusters. In the course of sequence comparisons, we found that the seemingly distant subclusters in *A. pachychristatus* NRRL 11440, *Ecd* and *Hty*, can be assembled into a single sequence (*Ecd/Hty*). The overlap of both sequences was confirmed by PCR. Moreover, *Ecd*/*Hty* was found to be virtually identical to echinocandin B biosynthesis-cluster *AE* which was deposited at the NCBI as a sequence from *A. delacroxii* NRRL 3860. To scrutinize this unexpected finding, genomic DNA from this strain and *A. pachychristatus* NRRL 11440 was amplified by PCR and sequenced. The calmodulin marker sequences of both strains were very similar, but not entirely identical (2 different bp). BLAST search and phylogenetic analysis showed that both of them were most similar to calmodulin sequences from other *A. pachycristatus* strains (99–100 % identity), and they clearly differed from those of *A. delacroxii* strains (92–94 % identity). The sequence of a PCR product from a section of the echinocandin gene cluster in *A. delacroxii* was very similar (97 % identity), but clearly distinct from the corresponding sequence of *A. pachycristatus* (≙ *AE*). Therefore, we suppose that *AE* originates from *A. pachycristatus* NRRL 11440 and not *A. delacroxii* NRRL 3860. Moreover, the partial sequence of the calmodulin gene and the remarkably high similarity of the gene cluster fragments strongly suggest that strain NRRL 3860 belongs to the *A. pachychristatus*/*A. rugulosa* group rather than to *A. delacroxii*. An in-depth examination of this strain will be necessary to clarify the taxonomic status.

## Methods

*Aspergillus nidulans* NRRL 8112 (= ATCC 58396) and *A. pachycristatus* NRRL 11440 (= *A. nidulans* var. *roseus* ATCC 58397) were purchased from LGC Standards/ATCC (UK). *A. delacroxii* NRRL 3860 (= *E. nidulans* var. *echinulata*) was obtained from the Agricultural Research Service Collection (ARS) of the United States Department of Agriculture (USDA), Peoria IL (USA).

The genome of *A. nidulans* NRRL 8112 was sequenced and assembled by the De Novo Sequencing Service of BaseClear (Netherlands) using Illumina technique. (Assembly statistics: N50: 65,796 bp, L50: 138, largest contig: 462,827 bp). The paired-end library was deposited at the NCBI in Bioproject PRJNA89151 SRA experiment SRX128799. The genome was annotated with the automatic gene prediction tool AUGUSTUS [23] and the “Antibiotics and Secondary Metabolite Analysis Shell” (antiSMASH) [24].

For PCR-experiments genomic DNA from *A. pachycristatus* NRRL 11440 and *A. delacroxii* NRRL 3860 was prepared as follows: A culture was grown from a spore suspension in potato dextrose medium (250 mL) at 24 °C and 160 rpm for 14 days. The mycelium was harvested, frozen in liquid nitrogen and ground to a fine powder. The DNA was then extracted as per GenElute™ Plant Genomic DNA Miniprep Kit (Qiagen, Germany).

PCR experiments were conducted as per NEB protocol using Phusion™ Master Mix. The following profile was used for amplification: 96 °C for 10 s (hot start), pause and addition of polymerase; 96 °C for 5 min; then 30 cycles at 96 °C for 30 s; 57 °C (T_M_-2) for 20 s; 72 °C for 2 min; and a final extension at 72 °C for 10 min. For primer pair *WH2_fw* and *WH2_rv* a melting temperature of T_M_ *=* 53 °C was used. Primer sequences are given in Table S1 in Additional file 1.

DNA and protein sequences were edited, compared and visualized with the Geneious 8.1 software platform [25]. Sequence identities were determined by aligning each set of protein sequences with MUSCLE [26] implemented in Geneious 8.1. with the default settings. The global sequence identity of the clusters was determined through a concatenation of the alignments of all proteins which occur in all clusters.

## Abbreviations

The acronyms for the biosynthetic clusters are explained in Table 1.

 BLAST(n), basic local alignment search tool (nucleotide); FGSC, Fungal Genetics Stock Center; NCBI, National Center for Biotechnology Information; NRPS, nonribosomal peptide synthase; NRRL, Northern Regional Research Laboratory; PCR, polymerase chain reaction

## Additional file

Additional file 1: PCR experiments and sequence alignments. This file containes further information on the PCR-reactions, a sequence alignment of the gene clusters *Ecd*, *Hty* and *AE* with the PCR product from genomic *A. pachycristatus* DNA, sequences of amplified ITS and calmodulin regions, their alignment with published sequences and a comparison of PCR-product sequences from the clusters of *E. pachychristatus* and *E. delacroxii.* (DOCX 8989 kb)
