# Supplementary material for: Echinocandin B biosynthesis: a biosynthetic cluster from Aspergillus nidulans NRRL 8112 and reassembly of the subclusters Ecd and Hty from Aspergillus pachycristatus NRRL 11440 reveals a single coherent gene cluster
Source: BMC Genomics. 2016 Aug 8;17:570. doi: 10.1186/s12864-016-2885-x (PMC4977696; doi:10.1186/s12864-016-2885-x)
Supplement: Additional file 1: — PCR experiments and sequence alignments. This file containes further information on the PCR-reactions, a sequence alignment of the gene clusters Ecd, Hty and AE with the PCR product from genomic A. pachycristatus DNA, sequences of amplified ITS and calmodulin regions, their alignment with published sequences and a comparison of PCR-product sequences from the clusters of E. pachychristatus and E. delacroxii. (DOCX 8989 kb) [file 12864_2016_2885_MOESM1_ESM.docx]

Supplementary file 1

**PCR and sequence alignments**

**Contents**

**I. PCR-experiments Page**

- **Figure S1**. Assembly of *Ecd* and *Hty* with primer binding sites **2**
- **Figure S2**. Gel eletrophoresis of PCR products **2**
- **Table S1**. Primers used for PCR-experiments. **2**

**II. Alignment *Ecd*/*Hty***

- **Figure S3**. Sequence alignment of the gene clusters *Ecd*, *Hty* and *AE* together with the PCR product from genomic *A. pachycristatus* DNA. **3**–**6**

**III. ITS region**

- **Figure S4**. Partial sequences of the ITS-region in *A. delacroxii* and *A. pachycristatus*. **7**
- **Table S2.** Best hits of a BLAST search with the partial ITS sequence from *Aspergillus pachycristatus* NRRL 11440 **7**
- **Figure S5**. Alignment of the ITS regions from *A. delacroxii* and *A. pachycristatus* and the best hits of a BLASTn search in the NCBI nr-database. **8**–**9**

**IV. Calmodulin gene**

- **Figure S6**. Alignment of partial calmodulin gene sequencs from *A. pachycristatus* NRRL 11440 and *A.* *delacroxii* NRRL 3860 with deposited calmodulin sequences from strains of the same species. **10**
- **Figure S7.** Phylogenetic tree of *Aspergillus* strains based on the calmodulin marker sequence.  **11**
- **Figure S8**. Partial sequences of the calmodulin gene from *A. delacroxii* and *A. pachycristatus*. **12**
- **Figure S9.** Alignment of calmodulin gene sequences of *A. pachychristatus* NRRL 11440 (ATCC 58397) from differerent sources. **12**

**V. Cluster analysis *E. pachychristatus*, *E. delacroxii***

- **Figure S9**. Sequence alignment of the *AE*-cluster (NCBI record AB720074) with the PCR-products from *A. pachycristatus* NRRL 11440 and from *A. delacroxii* NRRL 3860. **13**
- **Additional literature 14**

**1. PCR-experiments**


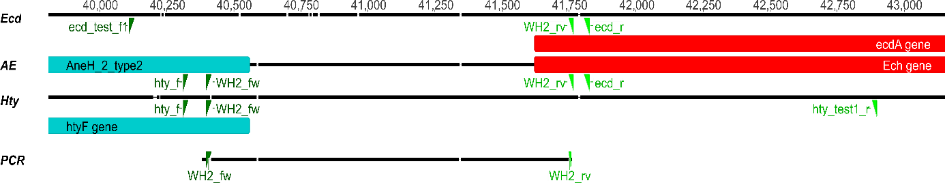


**Figure S1**. Assembly of *Ecd* and *Hty* with primer binding sites. *AE* was used as reference sequence. The PCR-product amplified from genomic *A. pachycristatus* DNA corresponds to *AE*, i.e., 5'-terminally to *Hty* and 3'-terminally to *Ecd*.


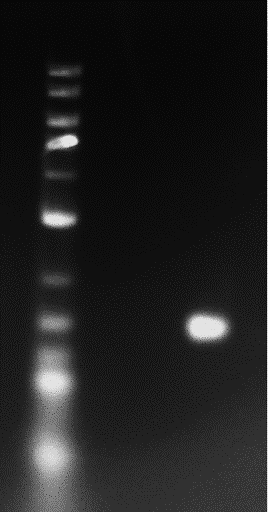


**1 2 3 4**

**1.5 kb**

**1 kb**

**3 kb**

**Figure S2**. Gel eletrophoresis of PCR products. lane 1: Marker 2log (NEB); lane 2: primers *ecd_test_f1* + *ecd_r* (expected size: 1.7 kb), lane 3: primers *hty_f* + *hty_test1_r* (expected size: 2.6 kb), lane 4: primers *hty_f* and *ecd_r* (expected size: 1.5 kb).

| **Name** | **Sequence** | **Direction** | **Target Sequence** | **position in original sequence** | |
| --- | --- | --- | --- | --- | --- |
|  |  |  |  | **Min** | **Max** |
| ***hty_test1_r*** | ATGCACTGCCCTTTGACAGA | reverse | *Hty (rev)* | 20,142 | 20,123 |
| ***hty_f*** | TGCGAAGGCGGTCTTAAACT | forward | *Hty (rev)* | 22,701 | 22,682 |
|  |  |  | *AE* | 21,888 | 21,907 |
| ***ecd_test_f1*** | CCCACAAACCCCGAAGCTAT | forward | *Ecd* | 39,769 | 39,788 |
| ***ecd_r*** | GCACACCGAATTACATCCGC | reverse | *AE* | 23,374 | 23,393 |
|  |  |  | *Ecd* | 41,449 | 41,468 |
| **WH2_fw** | AGCGCCACTGAACCATTC | forward | *Hty (rev)* | 24,901 | 24,918 |
|  |  |  | *AE* | 21,975 | 21,992 |
| **WH2_rv** | CGCAATGAGACGGATGGT | reverse | *Ecd* | 41,749 | 41,767 |
|  |  |  | *AE* | 41,767 | 41,749 |
| **ITS1** | TCCGTAGGTGAACCTGCGG | forward | *Ref. S1,10* |  |  |
| **ITS4** | TCCTCCGCTTATTGATATGC | reverse |  |  |  |
| **CMD5** | TTCCGCGCTTAACAGGACA | forward | *Ref. 10,22* |  |  |
| **CMD6** | CCGATAGAGGTCATAACGTGG | reverse |  |  |  |

**Table S1**. Primers used for PCR-experiments.

II. **Sequence alignments**

CLUSTAL

1

1 10 20 30 40 50 60

| | | | | | |

Eni GTAACTCCCTTGTCAGTTGGCGGCGCACGACATCGAACTGAAGCGGTATTGTCGCTGCAT

ecd GTGGCATTGGAGGGGGGTGTTGA**CCCACAAACCCCGAAGCTAT**TCCTGCCGGGCCATCAG

hty GTAACTCCCTTGTCAGTTGGCGGCGCACGACATCGAACTGAAGCGGTATTGTCGCTGCAT

PCR ----------------------------*ecd_test_f1*---------------------

Eni GGTGACCAAGAGCCCCCTGAGTGTAGTAGTTCTGAAGTTGCTCCTCGTGGATGATGTTAT

ecd TGTACGGCACTGCGCCTGCTGCATGTGCTAGATGATCTAGCTAG-CAAACCTACCATGGG

hty GGTGACCAAGAGCCCCCTGAGTGTAGTAGTTCTGAAGTTGCTCCTCGTGGATGATGTTAT

PCR ------------------------------------------------------------

Eni GGTCTAGGTCGGCATCTGGCTTTGCGAGAAATTCGCGCAATGAAGCTGGCGGCAGCACCA

ecd GATCTTTAACTTGTGAAGACTTTGTCAGCATTGATCATGTGGCAGTAGCCCCTTCTTAGG

hty GGTCTAGGTCGGCATCTGGCTTTGCGAGAAATTCGCGCAATGAAGCTGGCGGCAGCACCA

PCR ------------------------------------------------------------

Eni GCCAGGGTGGTTCCCCAATGTTTGGGAG**TGCGAAGGCGGTCTTAAACT**GGGAAAATCGGT

ecd AGTACCTGACCTGTAACCTGCCTTCTTAGACATGACAATATTAGACCACGACAATTTTAG

hty GCCAGGGTGGTTCCCCAATGTTTGGGAG**TGCGAAGGCGGTCTTAAACT**GGGAAAATCGGT

PCR ----------------------------------*hty_f*---------------------

Eni GAAATCCGTCCTCTACCATCTCGCGAACGCTGGTAACAATCCGTAGCTTGGTTCGAGCGC

ecd ATGATGATAATTTTACGCGCAATAGTTTTTAGGCCACGATAA-TACTTAACGATGACAAT

hty GAAATCCGTCCTCTACCATCTCGCGAACGCTGGTAACAATCCGTAGCTTGGTTCGAGCGC

PCR --------------------------------------ATCCGTAGCTTGGTTCGAGCGC

Eni CACTGAACCATTCGTCACGGACTCCAACCCACGGATGGGAGGCCCAGAATGCCCTGTGGG

ecd ATTATACCCATTCGGTATTATCACGGAGTTAAGATACTATCTCTGAGGTCCTCAAGTTGT

hty CACTGAACCATTCGTCACGGACTCCAACCCACGGATGGGAGGCCCAGAATGCCCTGTGGG

PCR CACTGAACCATTCGTCACGGACTCCAACCCACGGATGGGAGGCCCAGAATGCCCTGTGGG

Eni TGCGGGATCTGTTCAGCTGGATCACATAGCTGACCAGACTCACGGCGAGCACAACTGATG

ecd TCATGCTTGAGA--AGGATGTAGGTCAGCGGGGCTAGGCATCTAATTTCATTGAAGGCTG

hty TGCGGGATCTGTTCAGCTGGATCACATAGCTGACCAGACTCACGGCGAGCACAACTGATG

PCR TGCGGGATCTGTTCAGCTGGATCACATAGCTGACCAGACTCACGGCGAGCACAACTGATG

Eni CCGCAAGCAGGGCAGGTGTACTGTCTGTAGCGAGCATTGTTGAGGTTTTCCAGAGGAACA

ecd CTAGATTGTGAAAAACTACAAAATGTGGTTAGAGTTCATATGAAAAGAACATCTTTAACC

hty CCGCAAGCAGGGCAGGTGTACTGTCTGTAGCGAGCATTGTTGAGGTTTTCCAGAGGAACA

PCR CCGCAAGCAGGGCAGGTGTACTGTCTGTAGCGAGCATTGTTGAGGTTTTCCAGAGGAACA

Eni TGAGCGAGGAGGGTGTCTAACTTAACCATCCAGGTAGGCTACGCGGGCCATGGGATCGAA

ecd GCCAAATCCAGAAAGTGTGGCCTCCAGAAGTGTGGCCCTCAGGTGATGGAGCTTTGGTCT

hty TGAGCGAGGAGGGTGTCTAACTTAACCATCCAGGTAGGCTACGCGGGCCATGGGATCGAA

PCR TGAGCGAGGAGGGTGTCTAACTTAACCATCCAGGTAGGCTACGCGGGCCATGGGATCGAA

Eni TAATCAATCGAGCAGAGAGCGCGCCTCGCCAGTGTCAACGGATGACAGAGAGGATAAGAA

ecd TTGGCGCTTATGTACAAAGTAGAATGTCTTAACATTTTCTGA-GATAAATTCTAGCTCCC

hty TAATCAATCGAGCAGAGAGCGCGCCTCGCCAGTGTCAACGGATGACAGAGAGGATAAGAA

PCR TAATCAATCGAGCAGAGAGCGCGCCTCGCCAGTGTCAACGGATGACAGAGAGGATAAGAA

Eni GACATGGCCGGCATTGCTCAAAGATGAGACTGGCAACGTCAAATACATGTGCCGCGCCCT

ecd CCCAACTGATCCAAAAGGATACAGTAAAGTCAAATTCAACACTGAAGAAAACCATGCTAC

hty GACATGGCCGGCATTGCTCAAAGATGAGACTGGCAACGTCAAATACATGTGCCGCGCCCT

PCR GACATGGCCGGCATTGCTCAAAGATGAGACTGGCAACGTCAAATACATGTGCCGCGCCCT

Eni CTCACTCTGCGGTCGGTCGGGTCCTGAATTCCAAGCTGCCACAGAACAAGCATAATCTAT

ecd CTTTAAGGGCAAGCAAAAAGTTAATCCGCCAGAAGCGGGACCGTCGCAAAACCAGTCAAT

hty CTCACTCTGCGGTCGGTCGGGTCCTGAATTCCAAGCTGCCACAGAACAAGCATAATCTAT

PCR CTCACTCTGCGGTCGGTCGGGTCCTGAATTCCAAGCTGCCACAGAACAAGCATAATCTAT

Eni GCGCAGTCCATCAGATCATTATGACTACCACCCTTATGTTCTATCAATCCATTCCGTTTT

ecd GAAAAAATCATCCGAGTATAGCAAGATGTGTGGTGCTGATGTCTGTTTGGGCATACATAT

hty GCGCAGTCCATCAGATCATTATGACTACCACCCTTATGTTCTATCAATCCATTCCGTTTT

PCR GCGCAGTCCATCAGATCATTATGACTACCACCCTTATGTTCTATCAATCCATTCCGTTTT

Eni TACTCCAGCCAGTAGTTGTTACTAAGCAGCAGCACTAGCTGAGGAGCCTACAGCCTCCAA

ecd TAGGGAGAGTGGCCAAGTATACATTTTCTCTGCGCACGCTTCAGGTTTCTGGGCATTTAT

hty TACTCCAGCCAGTAGTTGTTACTAAGCAGCAGCACTAGCTGAGGAGCCTACAGCCTCCAA

PCR TACTCCAGCCAGTAGTTGTTACTAAGCAGCAGCACTAGCTGAGGAGCCTACAGCCTCCAA

Eni GGCGGAGCTGACTAACAATGCTGTTAGCCCTAACGTACTTCACCACCGA-----------

ecd GGGTTCGCAATTGGTACATTCCCCTGA---TAACGTACTTCACCACCGAACCGTCCAAAC

hty GGCGGAGCTGACTAACAATGCTGTTAGCCCTAACGTACTTCACCACCGA-----------

PCR GGCGGAGCTGACTAACAATGCTGTTAGCCCTAACGTACTTCACCACCGA-----------

Eni ------GCCGGCCAGACCGTATTGCATACAACTCGGCGGGTTTCGAACTGTGATATCTCA

ecd CACCGAGCCGGCCAGACCGTATTGCATACAAATTGGCGGGTTTCGAACTGTGATATCTCA

hty ------GCCGGCCAGACCGTATTGCATACAACTCGGCGGGTTTCGAACTGTGATATCTCA

PCR ------GCCGGCCAGACCGTATTGCATACAACTCGGCGGGTTTCGAACTGTGATATCTCA

Eni ACAACAACAGATTTAAATAAAGTCAAATATTACATGGAGAGTATTATAGCAATAAAAAAC

ecd ACAACAACAAATTTAAATAAAGTCAAATATTACATGGAGAGTATTATAGCAATAAAAAAC

hty ACAACAACAGATTTAAATAAAGTCAAATATTACATGGAGAGTATTATAGCAATAAAAAAC

PCR ACAACAACAGATTTAAATAAAGTCAAATATTACATGGAGAGTATTATAGCAATAAAAAAC

Eni TTGATGTCCAAGTTTATACGCCATTAGTCTTTCCGGTTCTTCTTATTACCGGGGAATCGA

ecd TTGATGTTTAAGTTTATATGCCATTAGTCTTTCCGGTTCTTCTTATTACCGGGGAATCGA

hty TTGATGTCCAAGTTTATACGCCATTAGTCTTTCCGGTTCTTCTTATTACCGGGGAATCGA

PCR TTGATGTCCAAGTTTATACGCCATTAGTCTTTCCGGTTCTTCTTATTACCGGGGAATCGA

Eni GTTATTACAGCAAGCATGTCTGTATTTATACTGGACCTAGTGGAGAAATGGATATAAAGC

ecd GGTATTACAGCAAGCATGTCTGTATTTATACTGGACCTAGTGGAGAAATGGATATAAAGC

hty GTTATTACAGCAAGCATGTCTGTATTTATACTGGACCTAGTGGAGAAATGGATATAAAGC

PCR GTTATTACAGCAAGCATGTCTGTATTTATACTGGACCTAGTGGAGAAATGGATATAAAGC

Eni GTTTAATGTCCTTTTACACAATATTAATAATTGATACTAACAAATATCTGGTGATTTCAG

ecd GTTTAATGTCCTTTTACACAATATTAATAATTGATACTAACAAATATCTGGTGATTTCAG

hty GTTTAATGTCCTTTTACACAATATTAATAATTGATACTAACAAATATTTGGTGGTCTCAG

PCR GTTTAATGTCCTTTTACACAATATTAATAATTGATACTAACAAATATCTGGTGATTTCAG

Eni GACGTTGTTGTTGAGATATCACAGTTCGAAACCCGCCGATTTGTATGCAATACGG-CTGG

ecd GACGTTGTTGTTGAGATATCACAGTTCGAAACCCGCCGATTTGTATGCAATACGG-CTGG

hty GACGTTGTTGTTGAGATATCGCAGTTCGAAAACTGCCAATTTGTATGCAATACGGTCTGG

PCR GACGTTGTTGTTGAGATATCACAGTTCGAAACCCGCCGATTTGTATGCAATACGG-CTGG

Eni CCGGCTCGGTGGTTTGGACGGTTCGGTGGTGAAGTACGTTAACTTCAGCCGGCCTCCAAG

ecd CCGGCTCGGTGGTTTGGACGGTTCGGTGGTGAAGTACGTTAACTTCAGCCGGCCTCCAAG

hty CCGGCTCGGTGGTTTGGACGGTTCGGTGGTGGAGTACGTTATGTATACCCCCCAACACGG

PCR CCGGCTCGGTGGTTTGGACGGTTCGGTGGTGAAGTACGTTAACTTCAGCCGGCCTCCAAG

Eni GCCTACTACACAGCCTCTGGTGAGACTATAGCGTCCAGTCAGCGCTAACGGCGAGCCCAA

ecd GCCTACTACACAGCCTCTGGTGAGACTATAGCGTCCAGTCAGCGCTAACGGCGAGCCCAA

hty CTGATTCGTCTAGGACTCCTACTACCCAACCCCACACCAACTCACAGATATGGAGATTAG

PCR GCCTACTACACAGCCTCTGGTGAGACTATAGCGTCCAGTCAGCGCTAACGGCGAGCCCAA

Eni TATAGCTTTTCAGACAGGAATATTGCCCCTTGTGTTATGACAGTTCCATCTCTCATGTTT

ecd TATAGCTTTTCAGACAGGAATATTGCCCCTTGTGTTATGACAGTTCCATCTCTCATGTTT

hty TAGTGTGGAAAAGTAGGGCCTGGTGGTGGCTCAGTCTTAGAAAGAACCAAAGCTGCCGTT

PCR TATAGCTTTTCAGACAGGAATATTGCCCCTTGTGTTATGACAGTTCCATCTCTCATGTTT

Eni CACTCTTGAATGACCTGTGCACTACCACTAACAACCCGACCATAACGCCAACAACTACAG

ecd CACTCTTGAATGACCTGTGCACTACCACTAACAACCCGACCATAACGCCAACAACTACAG

hty TGGAAAGCATCCGCTTACTCTAGTTCTAGGTGTTTAGCAGGCTTGTTGAACTTACTAAGT

PCR CACTCTTGAATGACCTGTGCACTACCACTAACAACCCGACCATAACGCCAACAACTACAG

Eni AAATCAGCGATAATAGATTTGTGACCGACGACATGCACAGAACCAACGAGATGGAACGGA

ecd AAATCAGCGATAATAGATTTGTGACCGACGACATGCACAGAACCAACGAGATGGAACGGA

hty TTCATGGTCACCCTATTTGCGCGACTGGAAGGCAATAAGGCATGTGTTGCGTTGCAGTCC

PCR AAATCAGCGATAATAGATTTGTGACCGACGACATGCACAGAACCAACGAGATGGAACGGA

dd

Eni AACGCGTATTTACAGAAATATGCGCGGAGGCATTGAAGGTCGAGATAGACGACTTGGACG

ecd AACGCGTATTTACAGAAATATGCGCGGAGGCATTGAAGGTCGAGATAGACGACTTGGACG

hty TAGGTACCTAAGATATCTATTTACTGCATCCTGGCCAGTCCAGGAAAGACAAATGAATAG

PCR AACGCGTATTTACAGAAATATGCGCGGAGGCATTGAAGGTCGAGATAGACGACTTGGACG

Eni AGACTCGCTCGTGGGTAGCACTGGGAGGCGACTCGATGGCTACCATCCGTCTCATTGCGC

ecd AGACTCGCTCGTGGGTAGCACTGGGAGGCGACTCGATGGCTACCATCCGTCTCATTGCGC

hty ACAAAGAATGAGAGAAGGCATTAACCTGCCTCCAAACACGAACTACGTATAAGTGTAGGA

PCR AGACTCGCTCGTGGGTAGCACTGGGAGGCGACTCGATGGCTACCATCCGTC---------

Eni GATGCGAGGAGCGCGGAATGAGGGCCAAGACG**GCGGATGTAATTCGGTGTGC**TAGCATTA

ecd GATGCGAGGAGCGCGGAATGAGGGCCAAGACG**GCGGATGTAATTCGGTGTGC**TAGCATTA

hty TGGCAGCCATGAGCAGAGACTATCCCCAGTAGAGCTCCTATTCACGTATA-CAAGCCATG

PCR --------------------------------------*ecd_r*-----------------

Eni CAGAGCTCTTCGAGACCATCCAGTATCTACAACCTTCCGAATCAATCGACCGCGAGGAAG

ecd CAGAGCTCTTCGAGACCATCCAGTATCTACAACCTTCCGAATCAATCGACCGCGAGGAAG

hty CATCTGACTGGAGTTGTTGAACTCCTCAATAAATTTATTGTTTGCTTTCATGGCCGTTGC

PCR ------------------------------------------------------------

Eni TCAAACCCGAGGATGCTGATGCAGCACCCTTTTCACTATGGCCTGAATACCACAATGCGA

ecd TCAAACCCGAGGATGCTGATGCAGCACCCTTTTCACTATGGCCTGAATACCACAATGCGA

hty CAGGTACTTGAAGGTGACTTGCATCTTTTCAACAGAAGCCAATGCTTGGGCGATGGCAGT

PCR ------------------------------------------------------------

Eni CGACGACAGAGGAGAAACAGAAGCTTTTAAACGAAGTGGCGAGGCACTGCAACTCCACTC

ecd CGACGACAGAGGAGAAACAGAAGCTTTTAAACGAAGTGGCGAGGCACTGCAACTCCACTC

hty AAGATTTTTGATCTAAGGCATTAGGTTAGCCTAGTGTCCAGAGATTGTCGAGGTGTTGTG

PCR ------------------------------------------------------------

Eni CCAATGATATTGAGGATGTTTATCCCTCCACACCATTACAAGAAGGCCTGATGGCCATCA

ecd CCAATGATATTGAGGATGTTTATCCCTCCACACCATTACAAGAAGGCCTGATGGCCATCA

hty ACTTGTCTCTCAGCCATTCAATTTCATTGACACCAGCCGCTCGAGTTCGACTCCTTTGAT

PCR ------------------------------------------------------------

Eni CCTCCCGGAGCCCCGCCGCCTATGTCGACCGTCGCGCT---TTTACCCTCCCCCCAACCG

ecd CCTCCCGGAGCCCCGCCGCCTATGTCGACCGTCGCGCT---TTTACCCTCCCCCCAACCG

hty GATAGCGGGGCCCTGAAAGGTGCCAACCTCTGCCAGGAAGCTTTTCCAGATATTCCGCCG

PCR ------------------------------------------------------------

Eni TTGATATTGCCCGGTTTCGAGCGGCGTTGGAAGCTCTCACGGCTAGAACGCACATTCTCC

ecd TTGATATTGCCCGGTTTCGAGCGGCGTTGGAAGCTCTCACGGCTAGAACGCACATTCTCC

hty TGCGTCATGGGTCAAATCGGGATACTTGATCTTTAGTTGGATTCGGCTCAGAATGCCTCG

PCR ------------------------------------------------------------

Eni GCACACGAATCATTATCGATCCTCTTAGCGGGCGATCGCTGCAAGTGGTGACCCGCAACG

ecd GCACACGAATCATTATCGATCCTCTTAGCGGGCGATCGCTGCAAGTGGTGACCCGCAACG

hty TCAAACTCCATGACACGGTTGGTGGTCAGAAAGAGGACCCCCTAGTAGTATTCAAGTTTC

PCR ------------------------------------------------------------

Eni AGGTGGTGTGGCGAGA---GGCAATGACACTAAATGACTATCTGGAGGACGATCGTCAAG

ecd AGGTGGTGTGGCGAGA---GGCAATGACACTAAATGACTATCTGGAGGACGATCGTCAAG

hty CGAAGGAAGACCATTACCAGGCAGTTGTGGCTACCTTGGCACAACACGCGATTCTCCATG

PCR ------------------------------------------------------------

Eni AAGGGATCGCACTCGGTCAGCCCTTGTCCCGGTGCGGTTTAATCCAAGATAAGGGGTCAG

ecd AAGGGATCGCACTCGGTCAGCCCTTGTCCCGGTGCGGTTTAATCCAAGATAAGGGGTCAG

hty AACACATCAGCTTCATCAATCAGAAGGATCGTGTTCAGGCGCTTCGCAATTTTAAAGATC

PCR ------------------------------------------------------------

Eni ACGGGGTGGAAGAAACTGTTTTTGTATGGACAGTGCACCACAGCATATACGACGGATGGA

ecd ACGGGGTGGAAGAAACTGTTTTTGTATGGACAGTGCACCACAGCATATACGACGGATGGA

hty TGATCAAGAGTCATCTCCAGTTGCAGGGGATTGCTCTGACCAGCTATTAATTCGCCCGCC

PCR ------------------------------------------------------------

Eni GCGCGCTACAACTCTACCGGCAGTTGGCCGCAATCTACAATTCCGAG--CAGCTATCCCC

cd GCGCGCTACAACTCTACCGGCAGTTGGCCGCAATCTACAATTCCGAG--CAGCTATCCCC

hty GAAACCTAACCCATGAGCACCCAACCGAAACA-TCTGCGGGATGGAAATCACCTACTGAA

PCR ------------------------------------------------------------

Eni TGTCGTCCCATATACGCGATTTGTGCGCTATCTACAGCAACAGGATCCCGACAGCGCGAC

ecd TGTCGTCCCATATACGCGATTTGTGCGCTATCTACAGCAACAGGATCCCGACAGCGCGAC

hty TAGAGAGGGTACCGAGATGCCTCTGCCTTCGCCTCGGCCGTCAAGGTCTTCCCAACTCCG

PCR ------------------------------------------------------------

Eni GCAATACTGG-CGAGACCAGCTCCAGGGGGAGGATATCATGGTGGATTGGCCGACTCTCC

ecd GCAATACTGG-CGAGACCAGCTCCAGGGGGAGGATATCATGGTGGATTGGCCGACTCTCC

hty GGAGGACCCCACCAGACCGAGTTGGTGCTAATTAATTCAGGCATGCAATTTTTTGAGGCT

PCR ------------------------------------------------------------

Eni CCACAGCAACATACCAACCCAGACCGCGTACCCAGTTCCAGGCCAATATCCTGCTCCCTG

ecd CCACAGCAACATACCAACCCAGACCGCGTACCCAGTTCCAGGCCAATATCCTGCTCCCTG

hty AACATAATCAGAGCCGGTAGTTCTAGGCAGAGCACCCGGTAGGGTTCCAGCCATGCTCTT

PCR ------------------------------------------------------------

Eni ATGTATCGGGCTCAGGGCTGGTG-ATGATGTCGGCTGTGCTTCGCGGAGCCTGGGCTTTA

ecd ATGTATCGGGCTCAGGGCTGGTG-ATGATGTCGGCTGTGCTTCGCGGAGCCTGGGCTTTA

hty AGAGTTTTGAGATTAA-CTGTTGCAAGAGAACTGCAGCTATCAATTCCCTGCCCCGGCAG

PCR ------------------------------------------------------------

Eni GTGATGGCCCAGTACAGCGGCTATAGTGATGTGATCTTTGGGGTTACCCTATCCGGCCGA

ecd GTGATGGCCCAGTACAGCGGCTATAGTGATGTGATCTTTGGGGTTACCCTATCCGGCCGA

hty CTGAAGACTAGCTACACTTCCAGCCCTTGC-TGCTCGCCGCTGCTCCGGAGAAGCTCTCT

PCR ------------------------------------------------------------

Eni AATGCCCCCGTCCCACAGGTTGCAGACATTACCGCTCCTCTGATAACGACGGTACCAGTA

ecd AATGCCCCCGTCCCACAGGTTGCAGACATTACCGCTCCTCTGATAACGACGGTACCAGTA

hty GACTTCCCGTACTCCTTCTTCACCTGCCAACTCAGGATACAAACGAAGCACGTCGTCTAG

PCR ------------------------------------------------------------

Eni CGGATCCGCGTGGACCAGAAGTTAACGGTGGCCGAGTTTTTGGATAGGATTCAAACACAA

ecd CGGATCCGCGTGGACCAGAAGTTAACGGTGGCCGAGTTTTTGGATAGGATTCAAACACAA

hty TTGTGGCATTGCAGCCCTGATATTTTCC**TCTGTCAAAGGGCAGTGCAT**CCTGACAAACTC

PCR ----------------------------------*hty_test1_r*---------------

Eni GCCACCGAGATGATAGAATACGAACATAC

ecd GCCACCGAGATGATAGAATACGAACATAC

hty CTCATGGATACTCTTAAAGTGAAATGCGG

PCR -----------------------------

**Figure S3**. Sequence alignment of the gene clusters *Ecd*, *Hty* and *AE* together with the PCR product from genomic *A. pachycristatus* DNA. Primer binding sites are indicated in bold. Sequence sections which belong to the echinocandin biosynthesis cluster are shaded.

> Aspergillus delacroxii NRRL 3860 partial ITS region (its1-its4)

GGCTGCCTCCGGGCGCCCACCTCCCACCCGTGACTACCTAACACTGTTGCTTCGGCGGGGAGCCCCCCAGGGGCGAGCCG

CCGGGGACCACTGAACTTCATGCCTGAGAGTGATGCAGTCTGAGCCTGAATACAAATCAGTCAAAACTTTCAACAATGGA

TCTCTTGGTTCCGGCATCGATGAAGAACGCAGCGAACTGCGATAAGTAATGTGAATTGCAGAATTCAGTGAATCATCGAG

TCTTTGAACGCACATTGCGCCCCCTGGCATTCCGGGGGGCATGCCTGTCCGAGCGTCATTGCTGCCCTCAAGCCCGGCTT

GTGTGTTGGGTCGTCGTCCCCCCCGGGGGACGGGCCCGAAAGGCAGCGGCGGCACCGTGTCCGGTCCTCGAGCGTATGGG

GCTTTGTCACCCGCTCGATTAGGGC

> Aspergillus pachycristatus NRRL 11440 partial ITS region (its1-its4)

GAGTGCGGGCTGCTCCGGGCGCCCACCTCCCACCCGTGACTACCTAACACTGTTGCTTCGGCGGGGAGCCCCCCAGGGGC

GAGCCGCCGGGGACCACTGAACTTCATGCCTGAGAGTGATGCAGTCTGAGCCTGAATACAAATCAGTCAAAACTTTCAAC

AATGGATCTCTTGGTTCCGGCATCGATGAAGAACGCAGCGAACTGCGATAAGTAATGTGAATTGCAGAATTCAGTGAATC

ATCGAGTCTTTGAACGCACATTGCGCCCCCTGGCATTCCGGGGGGCATGCCTGTCCGAGCGTCATTGCTGCCCTCAAGCC

CGGCTTGTGTGTTGGGTCGTCGTCCCCCCCGGGGGACGGGCCCGAAAGGCAGCGGCGGCACCGTGTCCGGTCCTCGAGCG

TATGGGGCTTTGTCACCCGCTCGATTAGGGCCGGCCGGGCGCCAGCCGGCGTCTCCAACCTTATTTTTCTCAGGTTGACC

TCGGATCAGGTAGGGATACCCGCTGAACTTAAGCATATCAATAAGCGGAGAGAAG

**Figure S4**. Partial sequences of the ITS-region in *A. delacroxii* and *A. pachycristatus*.

| **Description** | **Query cover** | **Ident** | **Accession** |
| --- | --- | --- | --- |
| Emericella sp. FppMV internal transcribed spacer 1, partial sequence; 5.8S ribosomal RNA gene and internal transcribed spacer 2, complete sequence; and 28S ribosomal RNA gene, partial sequence | 99% | 99% | [HQ647305.1](http://www.ncbi.nlm.nih.gov/nucleotide/314947162?report=genbank&log$=nucltop&blast_rank=1&RID=E6890XU201R) |
| Aspergillus nidulans strain QRF373 18S ribosomal RNA gene, partial sequence; internal transcribed spacer 1, 5.8S ribosomal RNA gene, and internal transcribed spacer 2, complete sequence; and 28S ribosomal RNA gene, partial sequence | 99% | 99% | [KP278174.1](http://www.ncbi.nlm.nih.gov/nucleotide/817011858?report=genbank&log$=nucltop&blast_rank=2&RID=E6890XU201R) |
| Aspergillus rugulosus NRRL 206 ITS region; from TYPE material | 99% | 99% | [NR_131290.1](http://www.ncbi.nlm.nih.gov/nucleotide/807045352?report=genbank&log$=nucltop&blast_rank=3&RID=E6890XU201R) |
| Aspergillus nidulans strain BPPTCC 6038 18S ribosomal RNA gene, partial sequence; internal transcribed spacer 1, 5.8S ribosomal RNA gene, and internal transcribed spacer 2, complete sequence; and 28S ribosomal RNA gene, partial sequence | 99% | 99% | [KP165435.1](http://www.ncbi.nlm.nih.gov/nucleotide/772252741?report=genbank&log$=nucltop&blast_rank=4&RID=E6890XU201R) |
| Aspergillus nidulans strain WM 11.60 isolate ISHAM-ITS_ID MITS259 18S ribosomal RNA gene, partial sequence; internal transcribed spacer 1, 5.8S ribosomal RNA gene, and internal transcribed spacer 2, complete sequence; and 28S ribosomal RNA gene, partial sequence | 99% | 99% | [KP131596.1](http://www.ncbi.nlm.nih.gov/nucleotide/731445086?report=genbank&log$=nucltop&blast_rank=5&RID=E6890XU201R) |
| Uncultured fungus clone L046938-122-076-B02-unis 18S ribosomal RNA gene, partial sequence; internal transcribed spacer 1, 5.8S ribosomal RNA gene, and internal transcribed spacer 2, complete sequence; and 28S ribosomal RNA gene, partial sequence | 99% | 99% | [JF289097.1](http://www.ncbi.nlm.nih.gov/nucleotide/354720354?report=genbank&log$=nucltop&blast_rank=6&RID=E6890XU201R) |
| Aspergillus nidulans strain NRRL 2395 18S ribosomal RNA gene, partial sequence; and internal transcribed spacer 1, 5.8S ribosomal RNA gene, internal transcribed spacer 2, and 28S ribosomal RNA gene, complete sequence | 99% | 99% | [AY373888.1](http://www.ncbi.nlm.nih.gov/nucleotide/34809368?report=genbank&log$=nucltop&blast_rank=7&RID=E6890XU201R) |
| Uncultured fungus clone CMH457 18S ribosomal RNA gene, partial sequence; internal transcribed spacer 1, 5.8S ribosomal RNA gene, and internal transcribed spacer 2, complete sequence; and 28S ribosomal RNA gene, partial sequence | 99% | 99% | [KF800548.1](http://www.ncbi.nlm.nih.gov/nucleotide/571431442?report=genbank&log$=nucltop&blast_rank=8&RID=E6890XU201R) |
| Emericella sp. FLN12d genomic DNA containing 18S rRNA gene, ITS1, 5.8S rRNA gene, ITS2 and 28S rRNA gene, strain FLN12d | 99% | 99% | [HG518551.1](http://www.ncbi.nlm.nih.gov/nucleotide/562814871?report=genbank&log$=nucltop&blast_rank=9&RID=E6890XU201R) |
| Emericella sp. FLN12c genomic DNA containing 18S rRNA gene, ITS1, 5.8S rRNA gene, ITS2 and 28S rRNA gene, strain FLN12c | 99% | 99% | [HG518550.1](http://www.ncbi.nlm.nih.gov/nucleotide/562814870?report=genbank&log$=nucltop&blast_rank=10&RID=E6890XU201R) |
| Emericella sp. FLN2c genomic DNA containing 18S rRNA gene, ITS1, 5.8S rRNA gene, ITS2 and 28S rRNA gene, strain FLN2c | 99% | 99% | [HG518510.1](http://www.ncbi.nlm.nih.gov/nucleotide/562814830?report=genbank&log$=nucltop&blast_rank=11&RID=E6890XU201R) |
| Emericella sp. FLN2b genomic DNA containing 18S rRNA gene, ITS1, 5.8S rRNA gene, ITS2 and 28S rRNA gene, strain FLN2b | 99% | 99% | [HG518509.1](http://www.ncbi.nlm.nih.gov/nucleotide/562814829?report=genbank&log$=nucltop&blast_rank=12&RID=E6890XU201R) |
| Aspergillus sp. FLN1a genomic DNA containing 18S rRNA gene, ITS1, 5.8S rRNA gene, ITS2 and 28S rRNA gene, strain FLN1a | 99% | 99% | [HG518504.1](http://www.ncbi.nlm.nih.gov/nucleotide/562814824?report=genbank&log$=nucltop&blast_rank=13&RID=E6890XU201R) |
| Emericella nidulans isolate B9 internal transcribed spacer 1, partial sequence; 5.8S ribosomal RNA gene and internal transcribed spacer 2, complete sequence; and 28S ribosomal RNA gene, partial sequence | 99% | 99% | [JN676111.1](http://www.ncbi.nlm.nih.gov/nucleotide/353441529?report=genbank&log$=nucltop&blast_rank=14&RID=E6890XU201R) |
| Emericella nidulans strain KCCM60326 18S ribosomal RNA gene, partial sequence; internal transcribed spacer 1, 5.8S ribosomal RNA gene, and internal transcribed spacer 2, complete sequence; and 28S ribosomal RNA gene, partial sequence | 99% | 99% | [HQ285615.1](http://www.ncbi.nlm.nih.gov/nucleotide/329458394?report=genbank&log$=nucltop&blast_rank=15&RID=E6890XU201R) |
| Uncultured Emericella clone AC_2d_B11 18S ribosomal RNA gene, partial sequence; internal transcribed spacer 1, 5.8S ribosomal RNA gene, and internal transcribed spacer 2, complete sequence; and 28S ribosomal RNA gene, partial sequence | 99% | 99% | [JF449463.1](http://www.ncbi.nlm.nih.gov/nucleotide/329184627?report=genbank&log$=nucltop&blast_rank=16&RID=E6890XU201R) |
| Aspergillus variecolor strain RGT-S7 18S ribosomal RNA gene, partial sequence; internal transcribed spacer 1, 5.8S ribosomal RNA gene, and internal transcribed spacer 2, complete sequence; and 28S ribosomal RNA gene, partial sequence | 99% | 99% | [HQ674656.1](http://www.ncbi.nlm.nih.gov/nucleotide/318101586?report=genbank&log$=nucltop&blast_rank=17&RID=E6890XU201R) |
| Emericella nidulans strain RGT-S3 18S ribosomal RNA gene, partial sequence; internal transcribed spacer 1, 5.8S ribosomal RNA gene, and internal transcribed spacer 2, complete sequence; and 28S ribosomal RNA gene, partial sequence | 99% | 99% | [HQ674655.1](http://www.ncbi.nlm.nih.gov/nucleotide/318101585?report=genbank&log$=nucltop&blast_rank=18&RID=E6890XU201R) |
| Emericella rugulosa isolate FppMV4 internal transcribed spacer 1, partial sequence; 5.8S ribosomal RNA gene and internal transcribed spacer 2, complete sequence; and 28S ribosomal RNA gene, partial sequence | 99% | 99% | [HQ647315.1](http://www.ncbi.nlm.nih.gov/nucleotide/314947172?report=genbank&log$=nucltop&blast_rank=19&RID=E6890XU201R) |
| Emericella sp. SS-S10 18S ribosomal RNA gene, partial sequence; internal transcribed spacer 1, 5.8S ribosomal RNA gene, and internal transcribed spacer 2, complete sequence; and 28S ribosomal RNA gene, partial sequence | 99% | 99% | [GU797140.1](http://www.ncbi.nlm.nih.gov/nucleotide/291361454?report=genbank&log$=nucltop&blast_rank=20&RID=E6890XU201R) |
| Aspergillus nidulans strain UOA/HCPF 10384 isolate ISHAM-ITS_ID MITS261 18S ribosomal RNA gene, partial sequence; internal transcribed spacer 1, 5.8S ribosomal RNA gene, and internal transcribed spacer 2, complete sequence; and 28S ribosomal RNA gene, partial sequence | 99% | 99% | [FJ878647.1](http://www.ncbi.nlm.nih.gov/nucleotide/237626012?report=genbank&log$=nucltop&blast_rank=21&RID=E6890XU201R) |
| Aspergillus nidulans strain UOA/HCPF 9186 isolate ISHAM-ITS_ID MITS268 18S ribosomal RNA gene, partial sequence; internal transcribed spacer 1, 5.8S ribosomal RNA gene, and internal transcribed spacer 2, complete sequence; and 28S ribosomal RNA gene, partial sequence | 99% | 99% | [FJ878641.1](http://www.ncbi.nlm.nih.gov/nucleotide/237626006?report=genbank&log$=nucltop&blast_rank=22&RID=E6890XU201R) |
| Emericella nidulans isolate NRRL 187 18S ribosomal RNA gene, partial sequence; internal transcribed spacer 1, 5.8S ribosomal RNA gene, and internal transcribed spacer 2, complete sequence; and 28S ribosomal RNA gene, partial sequence | 99% | 99% | [EF652427.1](http://www.ncbi.nlm.nih.gov/nucleotide/158535888?report=genbank&log$=nucltop&blast_rank=23&RID=E6890XU201R) |
| Emericella sp. HZ-17 18S ribosomal RNA gene, partial sequence; internal transcribed spacer 1, 5.8S ribosomal RNA gene, and internal transcribed spacer 2, complete sequence; and 28S ribosomal RNA gene, partial sequence | 99% | 99% | [EU301647.1](http://www.ncbi.nlm.nih.gov/nucleotide/162415954?report=genbank&log$=nucltop&blast_rank=24&RID=E6890XU201R) |
| Emericella nidulans strain RTMH13.C5 18S ribosomal RNA gene, partial sequence; internal transcribed spacer 1, 5.8S ribosomal RNA gene, and internal transcribed spacer 2, complete sequence; and 28S ribosomal RNA gene, partial sequence | 99% | 99% | [EU287942.1](http://www.ncbi.nlm.nih.gov/nucleotide/161789589?report=genbank&log$=nucltop&blast_rank=25&RID=E6890XU201R) |
| Emericella nidulans isolate F9 internal transcribed spacer 1, partial sequence; 5.8S ribosomal RNA gene and internal transcribed spacer 2, complete sequence; and 28S ribosomal RNA gene, partial sequence | 99% | 99% | [EF151434.1](http://www.ncbi.nlm.nih.gov/nucleotide/134143188?report=genbank&log$=nucltop&blast_rank=26&RID=E6890XU201R) |
| Emericella dentata genes for ITS1, 5.8S rRNA and ITS2, partial and complete sequence, strain: IFM 42024 | 99% | 99% | [AB248999.1](http://www.ncbi.nlm.nih.gov/nucleotide/119359842?report=genbank&log$=nucltop&blast_rank=27&RID=E6890XU201R) |
| Aspergillus rugulovalvus genes for ITS1, 5.8S rRNA and ITS2, partial and complete sequence, strain: IFM 54210 | 99% | 99% | [AB248977.1](http://www.ncbi.nlm.nih.gov/nucleotide/119359820?report=genbank&log$=nucltop&blast_rank=28&RID=E6890XU201R) |
| Emericella quadrilineata strain UWFP 613 18S ribosomal RNA gene, partial sequence; internal transcribed spacer 1, 5.8S ribosomal RNA gene, and internal transcribed spacer 2, complete sequence; and 28S ribosomal RNA gene, partial sequence | 99% | 99% | [AY213644.1](http://www.ncbi.nlm.nih.gov/nucleotide/37786125?report=genbank&log$=nucltop&blast_rank=29&RID=E6890XU201R) |
| Aspergillus nidulans strain PWQ2388 isolate ISHAM-ITS_ID MITS260 18S ribosomal RNA gene, partial sequence; internal transcribed spacer 1, 5.8S ribosomal RNA gene, and internal transcribed spacer 2, complete sequence; and 28S ribosomal RNA gene, partial sequence | 98% | 99% | [KP131594.1](http://www.ncbi.nlm.nih.gov/nucleotide/731445084?report=genbank&log$=nucltop&blast_rank=30&RID=E6890XU201R) |
| Emericella sp. AUMC 6937 18S ribosomal RNA gene, partial sequence; internal transcribed spacer 1, 5.8S ribosomal RNA gene, and internal transcribed spacer 2, complete sequence; and 28S ribosomal RNA gene, partial sequence | 99% | 99% | [JQ425379.1](http://www.ncbi.nlm.nih.gov/nucleotide/385258042?report=genbank&log$=nucltop&blast_rank=31&RID=E6890XU201R) |
| Emericella dentata isolate NRRL 4908 18S ribosomal RNA gene, partial sequence; internal transcribed spacer 1, 5.8S ribosomal RNA gene, and internal transcribed spacer 2, complete sequence; and 28S ribosomal RNA gene, partial sequence | 99% | 99% | [EF652488.1](http://www.ncbi.nlm.nih.gov/nucleotide/158535949?report=genbank&log$=nucltop&blast_rank=32&RID=E6890XU201R) |
| Emericella quadrilineata isolate NRRL 4581 18S ribosomal RNA gene, partial sequence; internal transcribed spacer 1, 5.8S ribosomal RNA gene, and internal transcribed spacer 2, complete sequence; and 28S ribosomal RNA gene, partial sequence | 99% | 99% | [EF652466.1](http://www.ncbi.nlm.nih.gov/nucleotide/158535927?report=genbank&log$=nucltop&blast_rank=33&RID=E6890XU201R) |
| Emericella sp. IFM 54245 genes for ITS1, 5.8S rRNA and ITS2, partial and complete sequence | 99% | 99% | [AB249018.1](http://www.ncbi.nlm.nih.gov/nucleotide/119359861?report=genbank&log$=nucltop&blast_rank=34&RID=E6890XU201R) |
| Emericella rugulosa genes for ITS1, 5.8S rRNA and ITS2, partial and complete sequence, strain: IFM 54242 | 99% | 99% | [AB249002.1](http://www.ncbi.nlm.nih.gov/nucleotide/119359845?report=genbank&log$=nucltop&blast_rank=35&RID=E6890XU201R) |
| Emericella dentata genes for ITS1, 5.8S rRNA and ITS2, partial and complete sequence, strain: IFM 42021 | 99% | 99% | [AB249000.1](http://www.ncbi.nlm.nih.gov/nucleotide/119359843?report=genbank&log$=nucltop&blast_rank=36&RID=E6890XU201R) |
| Emericella cleistominuta genes for ITS1, 5.8S rRNA and ITS2, partial and complete sequence, strain: IFM 48170 | 99% | 99% | [AB248989.1](http://www.ncbi.nlm.nih.gov/nucleotide/119359832?report=genbank&log$=nucltop&blast_rank=37&RID=E6890XU201R) |
| Emericella rugulosa genes for ITS1, 5.8S rRNA and ITS2, partial and complete sequence, strain: IFM 54234 | 98% | 99% | [AB248976.1](http://www.ncbi.nlm.nih.gov/nucleotide/119359819?report=genbank&log$=nucltop&blast_rank=38&RID=E6890XU201R) |
| Aspergillus sp. BAB-5562 18S ribosomal RNA gene, partial sequence; internal transcribed spacer 1, 5.8S ribosomal RNA gene, and internal transcribed spacer 2, complete sequence; and 28S ribosomal RNA gene, partial sequence | 99% | 99% | [KU504333.1](http://www.ncbi.nlm.nih.gov/nucleotide/973966921?report=genbank&log$=nucltop&blast_rank=39&RID=E6890XU201R) |
| Aspergillus sp. BAB-4413 18S ribosomal RNA gene, partial sequence; internal transcribed spacer 1, 5.8S ribosomal RNA gene, and internal transcribed spacer 2, complete sequence; and 28S ribosomal RNA gene, partial sequence | 99% | 99% | [KR154902.1](http://www.ncbi.nlm.nih.gov/nucleotide/820945312?report=genbank&log$=nucltop&blast_rank=40&RID=E6890XU201R) |

**Table S2**. Best hits of a BLAST search with the partial ITS sequence from *Aspergillus pachycristatus* NRRL 11440

**ITS region**
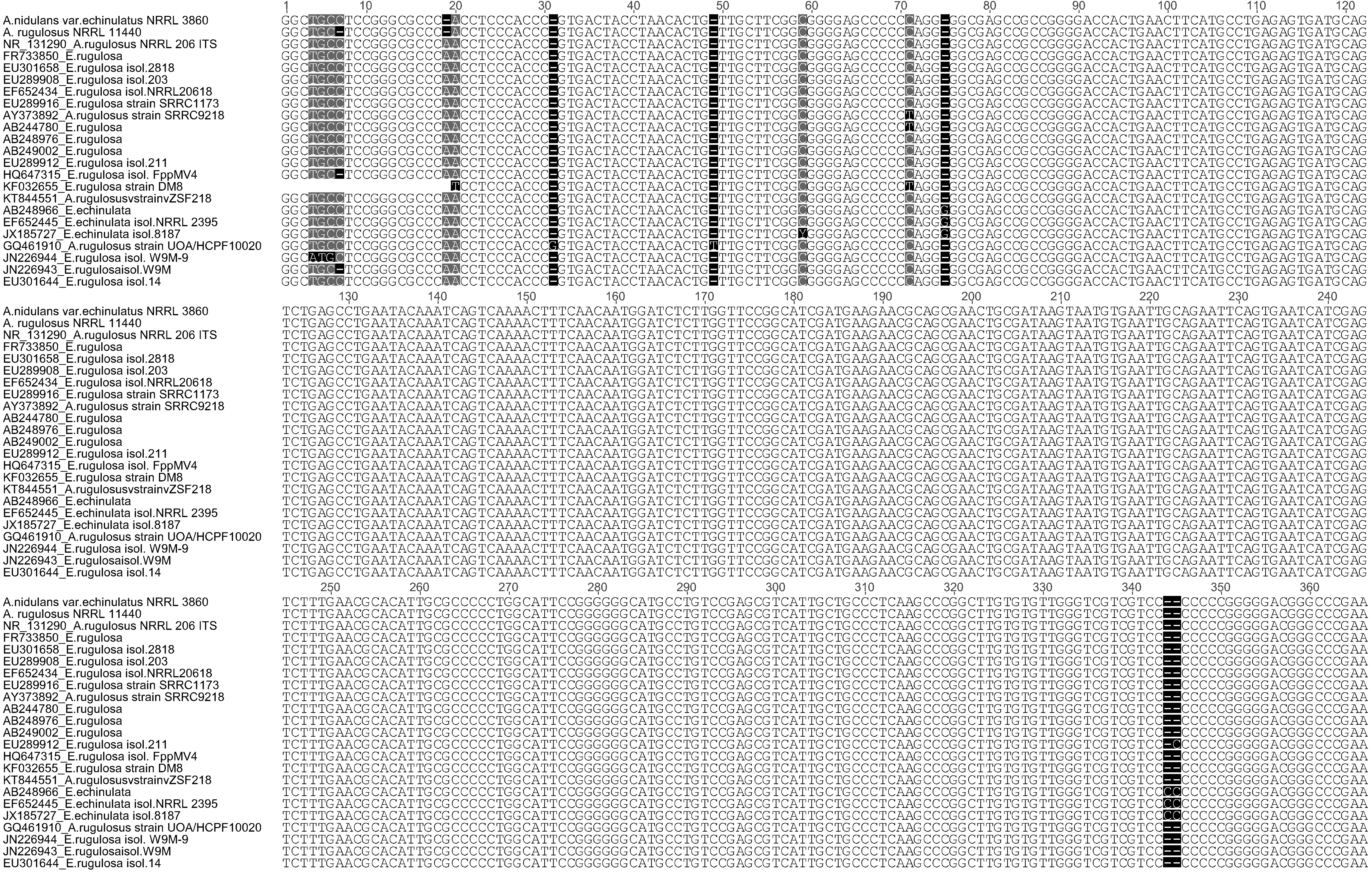


**
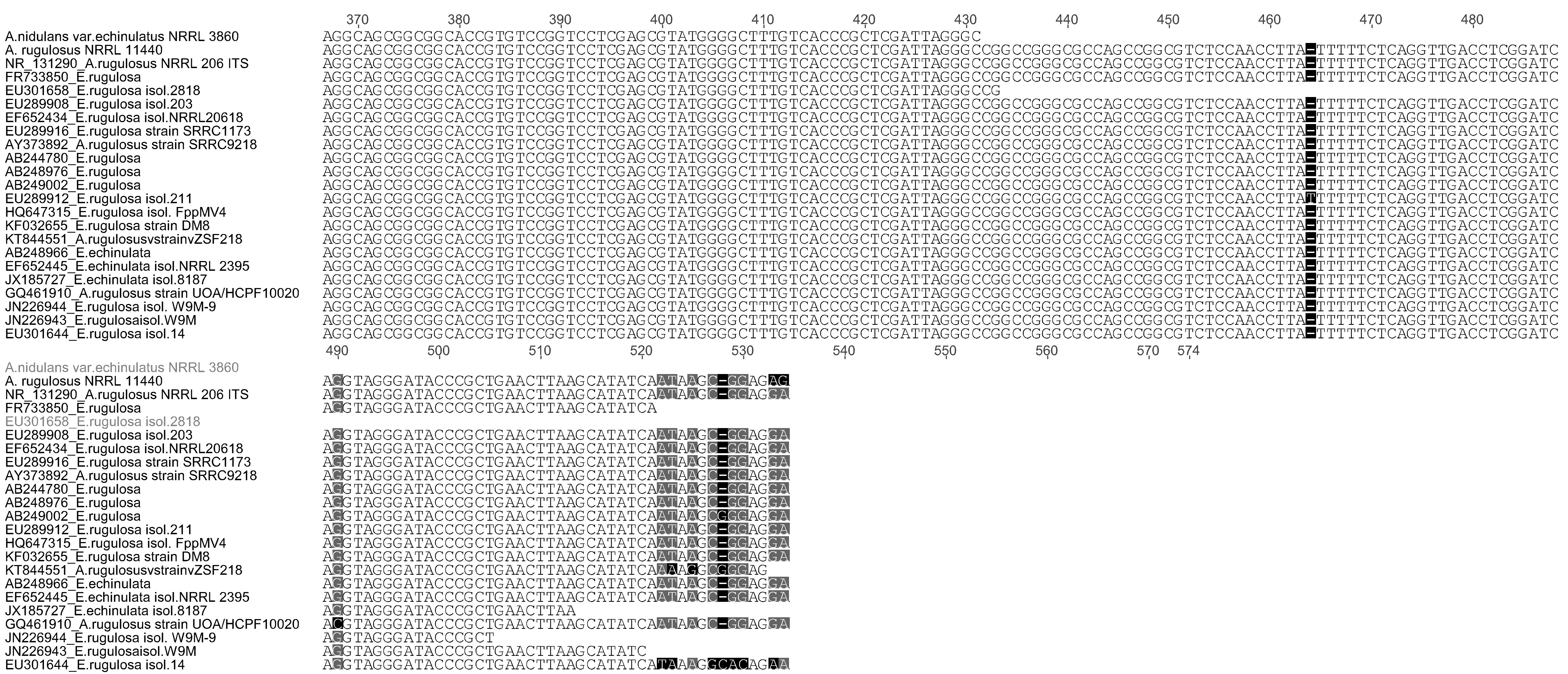
Figure S5**. Alignment of the ITS regions from *A. pachycristatus* NRRL 11440 (= *A. rugulosus*, NCBI Taxonomy ID: 41736) and *A. delacroxii* NRRL 11440 (= *E. nidulans* var echinulatus, *E. echinulata*, Taxonomy ID: 1810908) with ITS sequences of the same species (according to Taxonomy ID) from the NCBI Nucleotide database. The alignment starts about 20 bp after the ITS1-primer binding site, and terminates at the end of the ITS4-primer binding site.

**Calmodulin sequences**

**
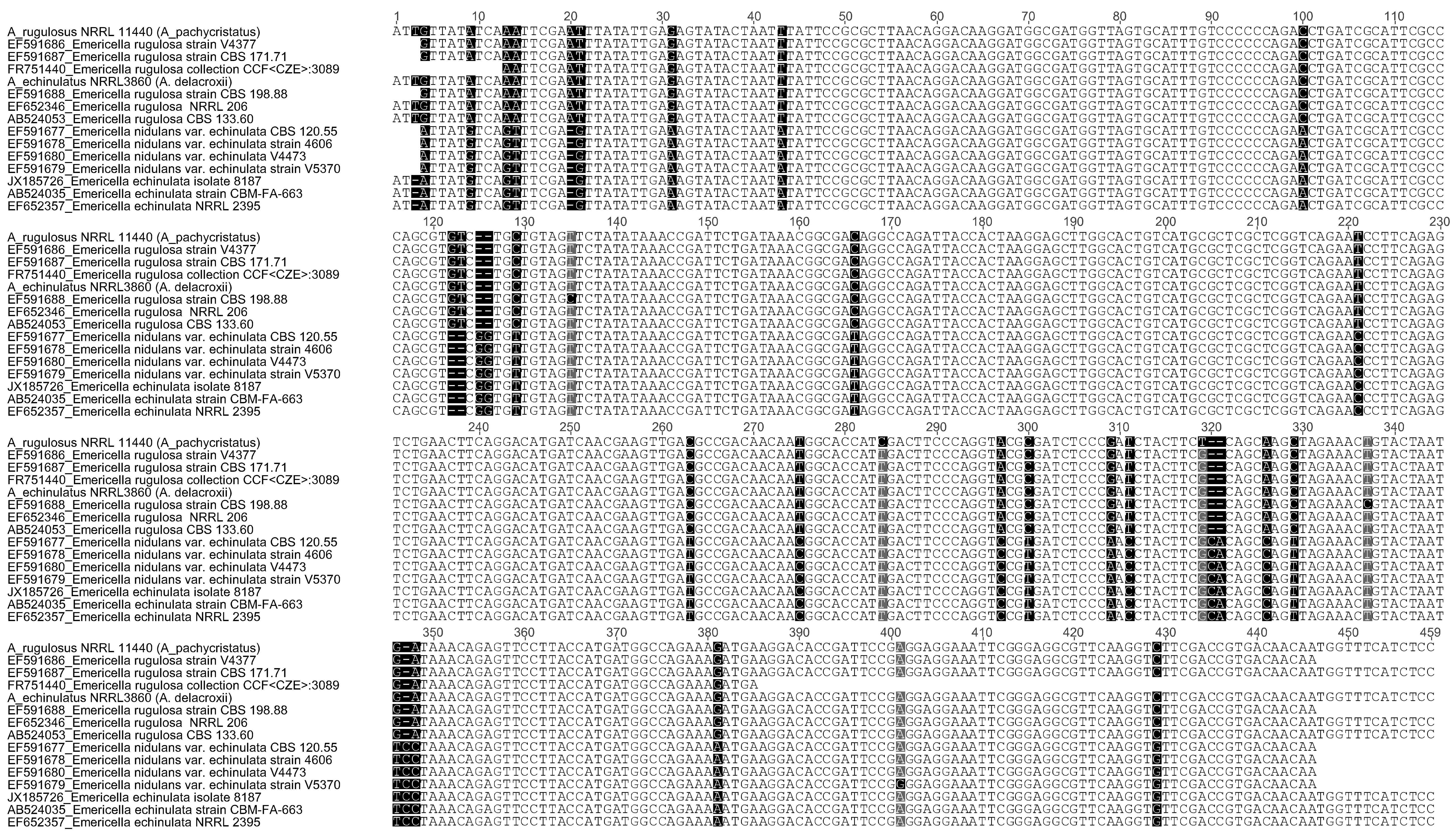
Figure S6**. Alignment of the partial calmodulin sequences from *A. pachycristatus* NRRL 11440 (= *A. rugulosus*, NCBI Taxonomy ID: 41736) and *A. delacroxii* NRRL 3860 (= *E. nidulans* var echinulatus, *E.* echinulata, Taxonomy ID: 1810908) with calmodulin sequences from strains of the same species (according to Taxonomy ID) from the NCBI Nucleotide database. The alignment starts about 25 bp after the CMD5-primer binding site, and ends 14 bp before the CMD6-primer binding site.

**Figure S7**. Phylogenetic tree of *Aspergillus* (*Emericella*) strains based on the calmodulin marker sequence.

The calmodulin entries were obtained by BLASTn seach or NCBI-nr database using the calmodulin sequences of *A. pachychristatus* NRRL 11440 and *E. echinulata* isolate 8187 (NCBI accession No. JX185726) as probes. The best 100 hits of each search were combined and duplicates were removed. The 121 remaining calmodulin sequences were aligned with MUSCLE[28] at default settings implemented in Geneious 8.1 [27]. The phylogeny was deduced with the ML method in RAxML BlackBox[S2]. 100 Bootstrap replicates were calculated with default settings. The phylogenetic distances were taken from the best tree and visualized as midpoint rooted tree with MEGA 6.[S3]

*>Aspergillus pachycristatus* NRRL 11440 calmodulin gene (cmd), partial cds

TCCGAGTACAAGGAGGCCTTTTCCCTGTTTGTAAGTGCCATTGGTTATTGTTATATCAAATTCGAATTTATATTGAGAGT

ATACTAATTTATTCCGCGCTTAACAGGACAAGGATGGCGATGGTTAGTGCATTTGTCCCCCCAGACCTGATCGCATTCGC

CCAGCGTGTCTGCTGTAGTTCTATATAAACCGATTCTGATAAACGGCGACAGGCCAGATTACCACTAAGGAGCTTGGCAC

TGTCATGCGCTCGCTCGGTCAGAATCCTTCAGAGTCTGAACTTCAGGACATGATCAACGAAGTTGACGCCGACAACAATG

GCACCATTGACTTCCCAGGTACGCGATCTCCCGATCTACTTCGCAGCAAGCTAGAAACTGTACTAATGATAAACAGAGTT

CCTTACCATGATGGCCAGAAAGATGAAGGACACCGATTCCGAGGAGGAAATTCGGGAGGCGTTCAAGGTCTTCGACCGTG

ACAACAATGGTTTCATCTCCGCTGCTGAGCTGCGTCACGTTATGACCTCTATCGG

> *Aspergillus delacroxii* NRRL 3860 calmodulin gene (cmd), partial cds

CCGAGTACAAGGAGGCCTTTTCCCTGTTTGTAAGTGCCATTGGTTATTGTTATATCAAATTCGAATTTATATTGAGAGTA

TACTAATTTATTCCGCGCTTAACAGGACAAGGATGGCGATGGTTAGTGCATTTGTCCCCCCAGACCTGATCGCATTCGCC

CAGCGTGTCTGCTGTAGTTCTATATAAACCGATTCTGATAAACGGCGACAGGCCAGATTACCACTAAGGAGCTTGGCACT

GTCATGCGCTCGCTCGGTCAGAATCCTTCAGAGTCTGAACTTCAGGACATGATCAACGAAGTTGACGCCGACAACAATGG

CACCATCGACTTCCCAGGTACGCGATCTCCCGATCTACTTCTCAGCAAGCTAGAAACTGTACTAATGATAAACAGAGTTC

CTTACCATGATGGCCAGAAAGATGAAGGACACCGATTCCGAGGAGGAAATTCGGGAGGCGTTCAAGGTCTTCGACCGTGA

CAACAATGGTTTCATCTCCGCCGCTGAGCTGCGTCACGTTATGACCTCTATCGG

**Figure S8.** Partial sequences of the calmodulin gene from *A. delacroxii* and *A. pachycristatus* determined via sequencing of PCR products.

CLUSTAL W (1.8) multiple sequence alignment

PCR-product -------------------CCGAGTACAAGGAGGCCTTTTCCCTGTTTGTAAGTGCCATT

AB524071 TTGACCGAAGAGCAAGTTTCCGAGTACAAGGAAGCCTTTTCCCTGTTTGTAAGTGCCATT

HQ222837 ------------------------------------------------------------

PCR-product GGTTATTGTTATATCAAATTCGAATTTATATTGAGAGTATACTAATTTATTCCGCGCTTA

AB524071 GGTTATTGTTATATCAAATTCGAATTTATATTGAGAGTATACTAATTTATTCCGCGCTTA

HQ222837 -------------------------------TGAGAGTATACTAATTTATTCCGCGCTTA

PCR-product ACAGGACAAGGATGGCGATGGTTAGTGCATTTGTCCCCCCAGACCTGATCGCATTCGCCC

AB524071 ACAGGACAAGGATGGCGATGGTTAGTGCATTTGTCCCCCCAGACCTGATCGCATTCGCCC

HQ222837 ACAGGACAAGGATGGCGATGGTTAGTGCATTTGTCCCCCCAGACCTGATCGCATTCGCCC

PCR-product AGCGTGTCTGCTGTAGTTCTATATAAACCGATTCTGATAAACGGCGACAGGCCAGATTAC

AB524071 AGCGTGTCTGCTGTAGTTCTATATAAACCGATTCTGATAAACGGCGACAGGCCAGATTAC

HQ222837 AGCGTGTCTGCTGTAGTTCTATATAAACCGATTCTGATAAACGGCGACAGGCCAGATTAC

PCR-product CACTAAGGAGCTTGGCACTGTCATGCGCTCGCTCGGTCAGAATCCTTCAGAGTCTGAACT

AB524071 CACTAAGGAGCTTGGCACTGTCATGCGCTCGCTCGGTCAGAATCCTTCAGAGTCTGAACT

HQ222837 CACTAAGGAGCTTGGCACTGTCATGCGCTCGCTCGGTCAGAATCCTTCAGAGTCTGAACT

PCR-product TCAGGACATGATCAACGAAGTTGACGCCGACAACAATGGCACCATCGACTTCCCAGGTAC

AB524071 TCAGGACATGATCAACGAAGTTGACGCCGACAACAATGGCACCATCGACTTCCCAGGTAC

HQ222837 TCAGGACATGATCAACGAAGTTGACGCCGACAACAATGGCACCATCGACTTCCCAGGTAC

PCR-product GCGATCTCCCGATCTACTTCTCAGCAAGCTAGAAACTGTACTAATGATAAACAGAGTTCC

AB524071 GCGATCTCCCGATCTACTTCTCAGCAAGCTAGAAACTGTACTAATGATAAACAGAGTTCC

HQ222837 GCGATCTCCCGATCTACTTCTCAGCAAGCTAGAAACTGTACTAATGATAAACAGAGTTCC

PCR-product TTACCATGATGGCCAGAAAGATGAAGGACACCGATTCCGAGGAGGAAATTCGGGAGGCGT

AB524071 TTACCATGATGGCCAGAAAGATGAAGGACACCGATTCCGAGGAGGAAATTCGGGAGGCGT

HQ222837 TTACCATGATGGCCAGAAAGATGAAGGACACCGATTCCGAGGAGGAAATTCGGGAGGCGT

PCR-product TCAAGGTCTTCGACCGTGACAACAATGGTTTCATCTCCGCCGCTGAGCTGCGTCACGTTA

AB524071 TCAAGGTCTTCGACCGTGACAACAATGGTTTCATCTCCGCCGCTGAGCTGCGTCACGTCA

HQ222837 TCAAGGTCTTCGACCGTGACAACAATGGTTTCATCTCCGCCGCTGAGCTGCGCCACGTTA

PCR-product TGACCTCTATCGG--------------------------------------

AB524071 TGACCTCTATCGGTGAGAAGCTCACCGATGACGAAGTCGACGAGATGATAC

HQ222837 TGACCTCTATCGG--------------------------------------

**Figure S9.** Alignment of calmodulin gene sequences of *A. pachychristatus* NRRL 11440 (ATCC 58397) from differerent sources. Two sequences of this gene have been deposited at the NCBI nucleotide database (see also Ref.[8]). NCBI Accession No: AB524071, HQ222837. The alignment shows that the sequences are not entirely identical. In this study PCR-product was used for sequence comparisons.

**Echinocandin biosynthesis**
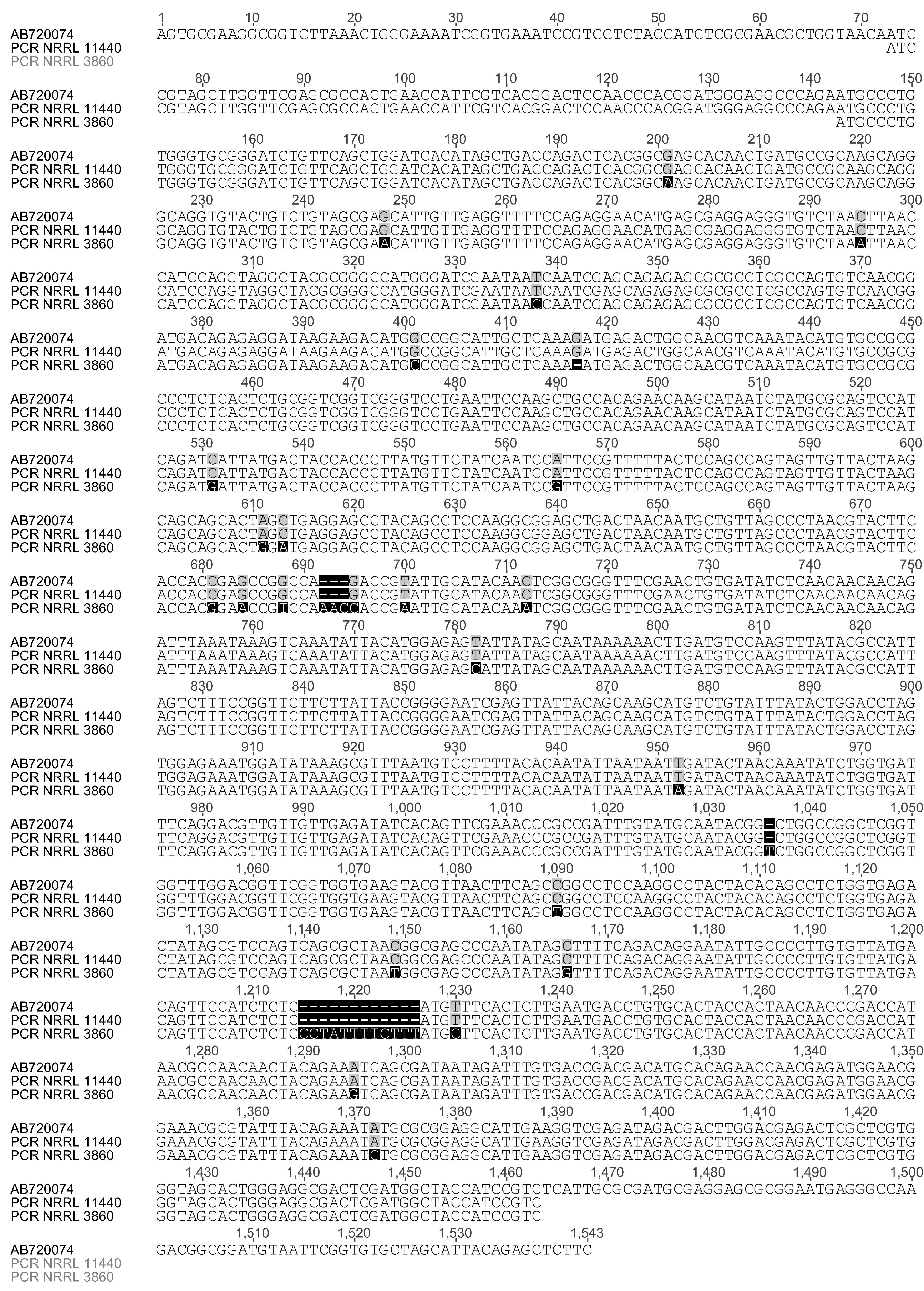


**Figure S10**. Sequence alignment of the *AE*-cluster (NCBI record AB720074) with the PCR-products from *A. pachycristatus* NRRL 11440 and from *A. delacroxii* NRRL 3860.The primer pairs hty_f/*ecd_r* and *WH2_fw/WH2_rv* were used, respectively. The WH2 primers were designed to amplify genomic DNA of non-sequenced *Aspergillus* strains without use of degenerate primers. They bind on a region which is entirely conserved in all characterized echinocandin gene clusters from *Aspergillus* species.

**Additional literature**

S1. White TJ, Bruns T, Lee S, Taylor J. Amplification and direct sequencing of fungal ribosomal rna genes for phylogenetics. In: PCR Protocols. San Diego: Academic Press; 1990: 315–22.

S3. Stamatakis A, Hoover P, Rougemont J: A Rapid Bootstrap Algorithm for the RAxML Web Servers. Syst Biol 2008;57(5):758–71.

S4. Tamura K, Stecher G, Peterson D, Filipski A, Kumar S. MEGA6: Molecular Evolutionary Genetics Analysis Version 6.0. Mol Biol Evol. 2013:30(12):2725–9.
